# The relation of alexithymia and attachment with type 1 diabetes management in adolescents: a gender-specific analysis

**DOI:** 10.1186/s40359-020-00396-3

**Published:** 2020-04-06

**Authors:** Zeinab Shayeghian, Mina Moeineslam, Elnaz Hajati, Mehrdad Karimi, Golshan Amirshekari, Parisa Amiri

**Affiliations:** 1 Research Center for Social Determinants of Health, Research Institute for Endocrine Sciences, Shahid Beheshti University of Medical Sciences, P.O.Box: 19395-4763, Tehran, Iran; 2 Department of Epidemiology and Biostatistics, School of Public Health, Tehran University of Medical Sciences, Tehran, Iran

**Keywords:** Type1 diabetes, Alexithymia, Attachment, HbA1C, Self-care, Adolescents

## Abstract

**Background:**

Several studies indicate the role of psychosocial factors in the management and control of chronic diseases in adolescents. In this regard, the roles of attachment and alexithymia in the management of type 1 diabetes in adolescents and related gender-specific patterns have rarely been the focus of empirical research. In this study we investigate the gender-specific relationship of alexithymia and attachment with self-care and blood glucose level in adolescents with type1 diabetes.

**Methods:**

This is a cross-sectional study conducted on adolescents aged 12–18 years, with type 1 diabetes. Participants were recruited from diabetes clinics and the Iranian Diabetes Society. Data were collected using the Farsi versions of the Toronto Alexithymia Scale (FTAS-20), the Inventory of Parent and Peer Attachment (IPPA) and the Summary of Diabetes Self-Care Activities Scale (SDSCA). Blood glucose levels were measured by determining HbA1c which were abstracted from medical records. Data were analyzed using SPSS21 software.

**Results:**

Participants were 150 adolescents (57% female), mean age 14.97 ± 2.30. Alexithymia (β = 0.10, *P* = 0.01), difficulty identifying feelings (β = 0.15, *P* = 0.03) and communication with mothers (β = − 0.08, *P* = 0.03) predicted HbA1c in girls, whereas no significant relationships were observed for HbA1c with alexithymia and attachment in boys. Factors that predicted self-care in girls were alexithymia (β = − 0.04, *P* = 0.02), difficulty identifying feelings (β = − 0.06, *P* = 0.04); in boys however in addition to these two factors predicting self-care [alexithymia (β = − 0.07, *P* = 0.01) and difficulty identifying feelings (β = − 0.11, *P* = 0.01)], we also found difficulty describing feelings (β = − 0.16, *P* = 0.02), communication with mother (β = 0.04, *P* = 0.04), alienation to mother (β = − 0.06, *P* = 0.03), to father (β = − 0.06, *P* = 0.01) and to peers (β = − 0.09, *P* = 0.03).

**Conclusions:**

Our results suggest that, in a gender-specific pattern, alexithymia and attachment could affect self-care and blood glucose level in adolescents with type 1 diabetes; findings that can be used to facilitate more effective treatment strategies and interventions in this age group.

## Background

Type 1 diabetes is one of the costly chronic diseases of childhood and adolescence that often is related with serious complications such as cardiovascular disorders and nephropathy [1]. The disease accounts for merely 5–10% of the population with diabetes [2] and according to the report of the International Diabetes Federation (IDF), of the estimated 497,100 children with type 1 diabetes, 64,000 are living in the Middle East and North Africa regions [3]. Statistics show that in Iran, the annual incidence of type 1 diabetes is estimated to be 3.7 per 100,000 people with a peak between 10 to 14 years of age [4]. Considering the globally rising trend in the number of people suffering from diabetes [2], current therapies for this disease indicate the need for major changes in the concepts of its management and treatment [5]. Evidence shows that self-management of chronic disorders including diabetes, can reduce patients’ dependence on health care services, increase their ability to identify their symptoms and thereby improving treatment outcomes [6].

As a result of cognitive and changes in psychological development during adolescence [7], passing responsibility from parents to adolescents [8] makes it even more complicated for the adolescents to control their diabetes. They have to learn self-care skills including blood glucose monitoring, identifying hypoglycemia and hyperglycemia, diet and exercise adherence and also insulin administration [8]. Since adolescents with diabetes are affected earlier by the disease, identifying the psychological factors affecting glycemic control is vital to optimum treatment and reducing complications of the disease [9]. Studies have shown that various psycho-social factors including effective coping [5], self-efficacy [10], expectations of positive outcomes [11], socioeconomic status [9], family functioning [8], and emotional regulation [12] can affect self-care and adherence in adolescents with diabetes.

Since emotional regulation is associated with better management of diabetes and a healthier lifestyle [12], alexithymia, a construct believed to be in conflict with emotional regulation, adversely affects poor self-care in these individuals and it as a risk factor for diabetes mellitus in the metabolic syndrome [13]. Alexithymia is defined as the disability and difficulty in cognitive processing of emotional information and regulation; its salient features were defined as: difficulty of identifying feelings (DIF), difficulty describing feelings (DDF), and externally oriented thinking (EOT). Persons with alexithymia are more at risk of poor glycaemic control [14] and they suffer from impaired interoception (disability in interpreting physical signals from the body when identifying one’s own emotions) and have difficulty distinguishing emotions from bodily sensations [15], deficiencies which could interfere with better management of the diabetes [14].

Another important factor in controlling type 1 diabetes in children is family functioning (specific to diabetes care). For example, positive parental emotional support is associated with improved metabolic control [16]. Research shows that attachment plays a major role in the development of emotion competence [17] and was associated with alexithymia in male patients [18]. The attachment theory proposes that threat and distress activate the attachment system (care-seeking); in fact the teenagers’ attachment style is an aid to predict how to deal with stress, regulate emotions, and change the health-related behaviors throughout a lifelong disease management process [19]. Attachment security depends on the quality of child care provided by parents, and over time, becomes the ‘internal working models’ of relationships in individuals [20]. People with insecure attachment styles, because of the adverse experiences with their attachment figures, have difficulties accepting the support they need to achieve the maximum self-care behaviors [21]. Accordingly, when confronted with the disturbance caused by the disease, individuals with diabetes face hardships in receiving support from others because they find it difficult to cope with intimate relationships, for managing dependency and developing trust [22]. Evidence shows a negative correlation between insecure attachment style and self-care (such as nutrition, exercise, and foot-care) in combating chronic diseases [21, 23, 24] and high levels of blood glucose in patients with type one and two diabetes [25].

In Iran, although researchers have investigated the relationships of alexithymia [26, 27] and attachment [28, 29] with blood glucose control in those with diabetes, limited studies have focused on adolescents. Furthermore, evidence shows that adherence and metabolic control are poorer in girls than boys [30], probably due to the differences in prevalence of alexithymia [31] and the different patterns of parent-adolescent relationships between genders [32]. To examine this hypothesis and to clarify the psychological dimensions of the management of diabetes type 1 in an Eastern-Mediterranean population, the current study aimed to investigate the gender-specific relationship of alexithymia and attachment with self-care and blood glucose levels in a sample of Tehranian adolescents. As one of the first investigations on this subject, our results could prove useful in the improvement of strategies and interventions for diabetes management in the studied community and for future cross-cultural studies.

## Methods

### Participants

Adolescents, aged 12–18 years, with type 1 diabetes were recruited for the current cross-sectional study from diabetes clinics in Tehran and the Iranian Diabetes Society (in the city of Tehran, Islamic Republic of Iran) between February 2015 and January 2016. A written informed consent form was obtained from all adolescents and their parents. The study was approved by the ethics committee of the Research Institute of Endocrine Sciences (RIES), Shahid Beheshti University of Medical Sciences, Tehran, Iran (ethical approval code: 31ECRIES93/10/23).

### Measures

In the current study given the context and research outlined in the introduction, participants completed three following questionnaires: 1)(Toronto alexithymia scale, 2) Inventory of parent and peer attachment, and 3) Summary of Diabetes Self-Care Activities Scale.

#### Toronto alexithymia scale (TAS-20)

The TAS-20 scale consists of 20 items, representing three factors: Difficulty identifying feelings (DIF), difficulty describing feelings (DDF) and externally oriented thinking (EOT) [33]. For each item, responders are asked to rate their extent of agreement with the statement on a 3-point response scale (1 = not true, 2 = a bit true, 3 = true); the internal consistency, reliability and validity for this version, as a whole, have been shown to be good in over 20 versions in different languages [33–35]. Regarding the Farsi version of this scale (FTAS-20), the Cronbach’s alpha coefficient for alexithymia has been reported at 0.85 and for the three subscales of DIF, DDF and EOT, to be 0.82, 0.75 and 0.72, respectively; test-retest reliability for FTAS-20 and its subscales ranged from 0.80 to 0.87 [35]. This scale is one of the most commonly used measures of alexithymia. Because of low reliability of the EOT sub-scale in some previous studies conducted on children and adolescents [36–38], the 8 items of this subscale have been removed from the current statistical analysis.

#### Inventory of parent and peer attachment (IPPA)

The Inventory of Parent and Peer Attachment (IPPA), a self-report instrument was used to assess both affective and cognitive dimensions of attachment among adolescents. This questionnaire was designed to assess both affective and cognitive dimensions of attachment security and trust in the availability and responsiveness of attachment figures among adolescents; the IPPA consists of 75 items, 25 from each of the original subscales, i.e., trust, communication, and alienation. For each IPPA item, participants first assess attachment quality with parents and then with peers using 5-point Likert-scales (1 = never true or almost never true, 5 = always true or almost always true) [39]. Internal reliabilities of the original version, as measured by Cronbach’s Alpha obtained were 0.87 for attachment to mother, 0.89 for attachment to father and 0.92 for attachment to peers [40]. The test–retest reliability for a sample of adolescents over a three-week period was also reported with correlation coefficients ranging between 0.86 for peer attachment and 0.93 for parent attachment [39]. Validity and reliability of this scale have been confirmed in various studies conducted in Iranian populations [41–43].

#### Summary of diabetes self-care activities scale (SDSCA)

This self-administered scale measures self-care behaviors in diet, exercise, blood glucose monitoring, medication usage, foot care and smoking; respondents report the frequencies of activities over the past 7 days on a continuum ranging from 0 to 7 [44]. Toobert et al., in their research have reported acceptable validity and reliability in several studies [45]. The validity of the Farsi version of this scale has been confirmed in previous studies in Iranian population [46–48]. This scale was used to assess self-management in those with diabetes.

#### Glycated hemoglobin measurement (HbA1c)

Hemoglobin A1C provides an average of blood sugar levels over the past 2 to 3 months and is an important test that shows how well diabetes is being controlled [49]. Blood glucose and A1c goals below 7.5% are recommended for children and adolescents with type 1 diabetes [50]. In this study, HbA1c values were measured using the HPLC method and the DS5 HbA1c measurement system (Hb gold). In the current study the A1cs levels were determined based on a single value at the visit closest to when the questionnaires were administered.

### Statistical analysis

Frequencies (%) for categorical data and mean ± SD for continuous variables are reported as descriptive statistics. To compare means between girls and boys, the independent samples t-test was used. Simple and multiple regression analysis were conducted to evaluate the relationship between HbA1c and SCSDA (dependent variables) with alexithymia and attachment (independent variables). Normality of residuals and error-variance consistency were examined in regression analysis to hold these assumptions. IBM SPSS Statistics 23 was utilized for statistical analysis [51].

## Results

Demographic and clinical characteristics of study participants are shown in Table 1. Participants included 150 adolescents (57% girls) with type 1 diabetes and mean ± SD ages of 14.99 ± 2.24 and 14.95 ± 2.38 years in girls and boys respectively. Results showed that 51.2% of girls and 52.5% of boys had a familial history of diabetes and 23.3% of girls and 19% of boys reported medication usage (Non-insulin drugs). Regarding self-care scores and all self-care activities, except for sports, boys (3.68 ± 2.00) and girls (2.70 ± 2.02, *P* = 0.04), did not significantly differ between gender groups (*P* > 0.05) (Table 1).

**Table 1:** Socio-demographic and clinical characteristics of patients with T1DM (*n* = 150)

Variables	Girls *N* = 86	Boys *N* = 64	***P***-value
**Age**(y)	14.99 ± 2.42	14.95 ± 2.38	0.93
**Familial history of diabetes** (yes)	43 (51.2)	32 (52.5)	0.88
**Drug therapy** (yes)	20 (23.3)	12 (19)	0.68
**Illness** (yes)	18 (22)	12 (19)	0.82
**Diabetes duration**	63.41 ± 45.43	63.60 ± 51.68	0.79
**A1C**	8.48 ± 1.94	8.09 ± 1.87	0.22
**SCSDA**	19.82 ± 5.85	21.31 ± 5.35	0.11
*Diet*	3.38 ± 1.24	3.65 ± 1.20	0.18
*Exercise*	2.70 ± 2.02	3.68 ± 2.00	0.04
*Blood Sugar Testing*	4.74 ± 2.57	4.57 ± 2.60	0.69
*Foot Care*	2.76 ± 1.77	3.00 ± 1.82	0.42
*Medications*	6.17 ± 1.63	6.39 ± 1.26	0.38
*Smoking*	0.92 ± 0.40	0.93 ± 0.35	0.89

Data are reported as mean ± SD and Frequency (percent)

**Fig. 1:**
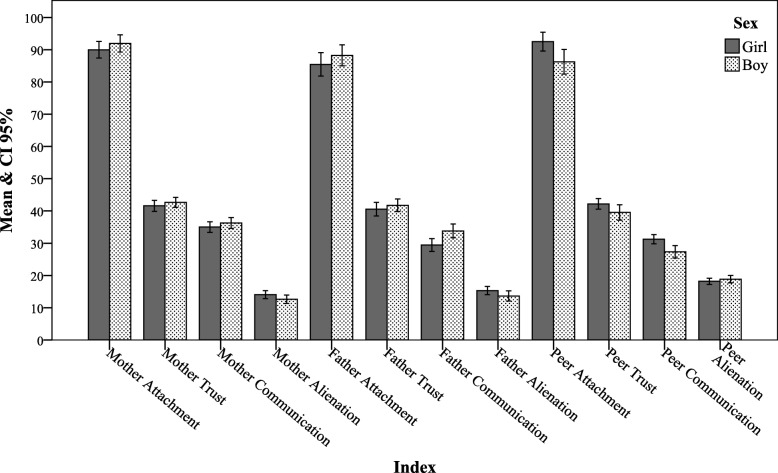
Comparison of genders on attachment (mother, father and peer). * P < 0.01, ** p < 0.05

As shown in Fig. 1, there were no significant difference between boys and girls regarding alexithymia and its subscales including IDF and DDF.

**Fig. 2:**
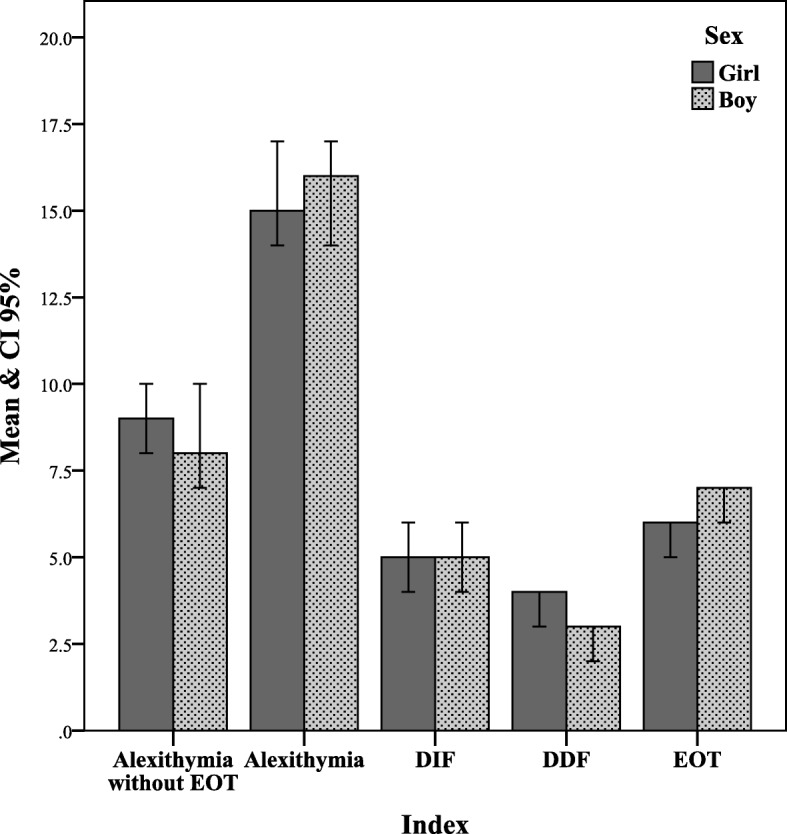
Comparison of genders based on alexithymia. * P < 0.01, ** p < 0.05

Figure 2 illustrates the means of total and subscales scores of attachment to mothers, fathers and peers (attachment, trust, communication and alienation). In terms of mother and father attachment, only father-communication differed significantly between gender groups (boys: 29.45 ± 8.94, girls: 33.81 ± 8.10, *P* = 0.04). Comparing the mean scores of peer attachment (boys: 86.27 ± 13.73, girls: 92.48 ± 12.51, *P* = 0.01) and peer-communication (boys: 31.24 ± 6.06, girls: 31.24 ± 6.06, *P* = 0.01) showed significant gender differences in these psychological aspects.

Table 2 shows the results of linear regression analysis in the association of HbA1c (as a dependent variable) with alexithymia and attachment (as independent variables). After adjusting for potential confounders including age, Body mass index (BMI) and parent history of diabetes, alexithymia (β = 0.09, SE: 0.04, *P* = 0.05) and DIF (β = 0.15, SE: 0.07, *P* = 0.03) significantly predict HbA1c only in girls. In terms of mother attachment, mother-communication (β = − 0.08, SE: 0.04, *P* = 0.03) had a negative effect on HbA1c in girls, but not in boys.

**Table 2:** Simple and multiple linear regression results (β (SE)) for predicting of A1C by alexithymia and attachment (mother, father and peer)

	Girls				Boys			
	unadjusted		adjusted		unadjusted		adjusted	
**Predictors**	β (SE)	P	β (SE)	P	β (SE)	P	β (SE)	P
**Alexithymia**								
*DIF*	0.16 (0.06)	0.01	0.15 (0.07)	0.03	−0.07 (0.07)	0.37	− 0.07 (0.10)	0.48
*DDF*	0.17 (0.08)	0.04	0.10 (0.09)	0.26	−0.10 (0.11)	0.35	−0.10 (0.14)	0.48
**total**	0.11 (0.04)	0.01	0.09 (0.04)	0.05	−0.04 (0.05)	0.33	−0.05 (0.06)	0.43
**Mother Attachment**								
*Mother-Trust*	−0.02 (0.03)	0.60	−0.03 (0.03)	0.31	−0.01 (0.04)	0.81	−0.04 (0.05)	0.36
*Mother-Communication*	−0.07 (0.03)	0.03	−0.08 (0.04)	0.03	−0.03 (0.04)	0.49	−0.02 (0.05)	0.69
*Mother-Alienation*	0.05 (0.04)	0.19	0.07 (0.05)	0.17	−0.02 (0.05)	0.65	0.01 (0.07)	0.86
**total**	−0.03 (0.02)	0.28	−0.03 (0.02)	0.22	0.001 (0.03)	0.98	0.001 (0.04)	0.99
**Father Attachment**								
*Father-trust*	−0.02 (0.02)	0.40	−0.02 (0.03)	0.44	−0.02 (0.03)	0.65	−0.04 (0.04)	0.35
*Father-communication*	−0.03 (0.02)	0.21	−0.04 (0.03)	0.21	−0.05 (0.03)	0.11	−0.04 (0.04)	0.23
*Father-Alienation*	0.06 (0.04)	0.13	0.07 (0.05)	0.17	0.002 (0.04)	0.97	0.03 (0.06)	0.62
**total**	−0.01 (0.02)	0.43	−0.02 (0.02)	0.30	−0.03 (0.02)	0.24	−0.02 (0.02)	0.37
**Peer attachment**								
*Peer-Trust*	−0.05 (0.03)	0.12	−0.01 (0.04)	0.69	0.004 (0.03)	0.89	0.01 (0.04)	0.69
*Peer-communication*	−0.05 (0.03)	0.12	−0.03 (0.03)	0.49	−0.03 (0.04)	0.42	−0.01 (0.04)	0.90
*Peer – Alienation*	−0.06 (0.05)	0.21	−0.10 (0.06)	0.10	0.02 (0.06)	0.74	0.05 (0.09)	0.55
**total**	−0.04 (0.02)	0.03	−0.04 (0.03)	0.15	0.001 (0.02)	0.97	0.01 (0.03)	0.57

Adjustment was conducted by age, BMI and parent history of diabetes

Results of simple and multiple linear regression analysis on the association of SCSDA (as a dependent variable) with alexithymia and attachment are presented in Table 3. The effects of alexithymia (β = − 0.04, SE: 0.02, *P* = 0.05) and DIF (β = − 0.06, SE: 0.03, *P* = 0.04) were significant in girls. In boys, the effects of alexithymia (β = − 0.08, SE: 0.03, *P* = 0.01), DIF (β = − 0.11, SE: 0.04, *P* = 0.01) and DDF (β = − 0.16, SE: 0.06, *P* = 0.02) were significant on the SCSDA. Also, mother-communication (β = 0.04, SE: 0.02, *P* = 0.04) and mother-alienation (β = − 0.06, SE: 0.03, *P* = 0.03) significantly predict SCSDA in boys. Also, for boys the negative effect of father-alienation (β = − 0.06, SE: 0.02, *P* = 0.01) and peer-alienation (β = − 0.09, SE: 0.04, *P* = 0.03) significantly reduced SCSDA, while in girls, none of the types of attachment were predictors of self-care.

**Table 3:** Simple and multiple regression results (β (SE)) for predicting of SCSDA by alexithymia and attachment (mother, father and peer)

	Girls				Boys			
	unadjusted		adjusted		unadjusted		adjusted	
**Predictors**	β (SE)	P	β (SE)	P	β (SE)	P	β (SE)	P
**Alexithymia**								
*DIF*	−0.07 (0.03)	0.02	−0.06 (0.03)	0.04	−0.03 (0.03)	0.40	−0.11 (0.04)	0.01
*DDF*	−0.09 (0.04)	0.03	−0.07 (0.04)	0.13	−0.02 (0.05)	0.66	−0.16 (0.06)	0.02
**total**	−0.05 (0.02)	0.01	−0.04 (0.02)	0.05	−0.02 (0.02)	0.47	−0.08 (0.03)	0.01
**Mother Attachment**								
*Mother-Trust*	0.02 (0.01)	0.27	0.001 (0.02)	0.99	0.04 (0.02)	0.04	0.04 (0.02)	0.07
*Mother-Communication*	0.03 (0.02)	0.06	0.01 (0.02)	0.45	0.05 (0.02)	0.02	0.04 (0.02)	0.04
*Mother-Alienation*	−0.04 (0.02)	0.03	−0.03 (0.02)	0.13	−0.04 (0.02)	0.12	−0.06 (0.03)	0.03
**total**	0.002 (0.01)	0.83	−0.01 (0.01)	0.49	0.02 (0.01)	0.06	0.01 (0.02)	0.44
**Father Attachment**								
*Father-trust*	0.03 (0.01)	0.004	0.02 (0.01)	0.08	0.03 (0.02)	0.05	0.02 (0.02)	0.25
*Father-communication*	0.04 (0.01)	0.002	0.02 (0.02)	0.19	0.03 (0.01)	0.03	0.02 (0.02)	0.15
*Father-Alienation*	−0.04 (0.02)	0.04	−0.04 (0.03)	0.20	−0.05 (0.02)	0.01	−0.06 (0.02)	0.01
**total**	0.02 (0.01)	0.003	0.01 (0.01)	0.17	0.02 (0.01)	0.01	0.01 (0.01)	0.60
**Peer attachment**								
*Peer-Trust*	0.03 (0.02)	0.04	0.01 (0.02)	0.50	0.01 (0.01)	0.32	0.01 (0.02)	0.61
*Peer-communication*	0.04 (0.02)	0.02	0.02 (0.02)	0.30	0.002 (0.02)	0.88	−0.004 (0.02)	0.84
*Peer – Alienation*	−0.004 (0.03)	0.86	−0.02 (0.03)	0.53	−0.04 (0.03)	0.21	−0.09 (0.04)	0.03
**total**	0.03 (0.01)	0.005	0.01 (0.01)	0.29	−0.01 (0.01)	0.58	−0.01 (0.01)	0.39

Adjustment was conducted by age, BMI and parent history of diabetes

## Discussion

To the best of our knowledge, this study is the first to examine the role of both attachment and alexithymia on diabetes management in adolescents. According to our findings, blood glucose levels in girls were directly associated with their psychological status; an association which in boys, was indirect via their self-care. In this regard, mothers play a unique role in determining the level of blood glucose in girls, while self-care in boys is also influenced by fathers and peers. Although blood glucose level was affected by alexithymia and DIF only in girls, self-care was influenced by these two factors in both genders and by DDF just in boys.

The current findings of the key role of mothers in predicting HbA1c levels in girls and self-care in boys are consistent with those documented in existing literature [52, 53]. Self-control is established in the context of early mother-child attachment, a relationship in the framework of which, higher skills of mothers in managing difficulties can lead to better self-control in adolescents [53]. Although communication with mothers does not increase self-care in girls with diabetes, it may reduce their HbA1c levels through other variables such as reducing anxiety and improving insulin regulation. This suggests that health-care professionals should pay specific attention to mother-daughter relationship and try to improve these relationships for achieving better glycemic control. In boys, communication with mother and alienation to parents and peers both have significant effects in the prediction of self-care. Apparently boys need more support resources and girls are more independent in their responsibilities to control diabetes [54]. Furthermore, although many studies have shown that peers have a greater impact on self-care in girls than in boys [55], results not consistent with those of the current study regarding diabetes control. Considering that exercise levels in boys are significantly higher than in girls and as boys usually exercise with their friends, it seems that the relationship with peers in boys contributes to improving their self-care via increased physical activity. Based on these findings, for improving self-care education in boys, it is recommended to consider the effect of social support. This finding implies that in boys, self-care trainings by peer groups could be more efficient.

Our sex-specific results regarding the diagnostic value of alexithymia and DIF in predicting the level of HbA1c in girls, but not in boys, are difficult to compare with those of other studies because there is no gender-specific study documented comparing this relationship; however two previous studies [14, 56] without any gender comparison showed similar findings. A possible hypothesis for this difference in the pattern of association of alexithymia and DIF with HbA1c in boys and girls could be that in girls with diabetes [30], having alexithymia [57] increases experiences of negative emotions which may be accompanied by increased HbA1c level. Since emotions affect blood glucose and the course of diabetes, an impact which may directly be a result of the release of stress hormones that interfere with insulin regulation [58].

Another, important findings on the predicting role of alexithymia in self-care of adolescents are consistent with results of other studies [26, 27]; however, gender-specific analysis showed that DIF affected self-care in both genders and DDF was a predictor of self-care only in boys. Individuals with alexithymia may be unaware of their physical symptoms when they need more insulin and, as a result, they overlook their abilities of self-care [59]. Also, since adolescents because of increased dopaminergic activity during puberty do not have the cognitive and emotional maturity of adults [7], their compliance with disease treatment could be weaker than adults. Furthermore, people, who are unable to identify their emotions and those of others have difficulty in building social relationships and accepting support from others [60]. Also regarding gender differences, in the relationship between subscales of alexithymia and self-care, one of the hypotheses could be that men tend to express themselves less emotionally and encounter more problems in getting social support [61]. While expressing emotions is closely related to receiving support of healthcare professionals and effectiveness of treatment [56], the ability of men in identifying and describing their feelings increases their self-care.

Regarding strengths, this study, for the first time, demonstrates gender differences in the relationship of alexithymia and attachment with HbA1c levels and self-care activities in adolescents with type 1 diabetes. Of limitations, although the parent and peer attachment scale has been used in many studies, it does have some conceptual limitations in identifying attachment styles. Using a more accurate instrument to assess different attachment styles (secure and insecure) in adolescents could facilitate a better understanding of relationships between attachment and management of illness. Also we based A1c levels on single results close to the time of the study visit rather than mean A1c based on several visits, which would have been more accurate, due to the unavailability of that data. Finally it was not possible to examine some other variables such as adolescents’ temperaments and parent’s attachment styles which could affect the relation of alexithymia and attachment with diabetes management in adolescents.

## Conclusions

The current findings demonstrate that alexithymia may interfere with self-care and blood glucose levels in adolescents with type 1 diabetes via a gender-specific pattern. While in girls more attention to mother-communication and alexithymia may result in more efficient diabetes management, focusing on fathers and peer relationships would be also important in boys. Hence, providing a gender-tailored relationship with parents and friends may result in better management of diabetes in this age group and could be useful for designing future health promotion strategies and interventions.

## Data Availability

The datasets used in the current study are available from the corresponding author on reasonable request.

## Abbreviations

FTAS-20: Farsi versions of the Toronto Alexithymia Scale
IPPA: Inventory of Parent and Peer Attachment
SDSCA: Summary of Diabetes Self-Care Activities Scale
HbA1c: Hemoglobin A1c
IDF: International Diabetes Federation
DIF: Difficulty of identifying feelings
DDF: Difficulty describing feelings
EOT: Externally oriented thinking
RIES: Research Institute of Endocrine Sciences
BMI: Body mass index
